# When It’s Not A Stroke After All: Atypical Presentation of Acute Aortic Dissection: A Case Report

**DOI:** 10.51894/001c.144605

**Published:** 2025-09-30

**Authors:** Jash Mody

**Affiliations:** 1 Department of Emergency Medicine Henry Ford Wyandotte

## 102

### INTRODUCTION

Acute aortic dissection is a life-threatening condition that typically presents with chest pain. However, atypical presentations may lead to diagnostic delays. We report a 64-year-old female presenting with acute right lower extremity pain and numbness, initially evaluated for stroke. Imaging revealed an acute Stanford Type A aortic dissection extending into the right lower extremity. This case highlights the need to consider vascular emergencies in atypical neurological presentations.

### CASE DESCRIPTION

A 64-year-old female with hypertension, depression, and carpal tunnel syndrome presented with sudden-onset severe right leg pain and numbness for 30 minutes. She denied chest pain, dizziness, or headache.

FINDINGS

- Vitals: BP 260/100 mmHg, HR 98 bpm, SpO2 100%.
- Neurologic: Right lower extremity sensory deficit and weakness.
- Cardiovascular: Diminished right lower limb pulses.

Given the stroke-like presentation, a stroke alert was activated. CT head and neck angiography were negative for stroke, but CT angiography of the chest, abdomen, and pelvis revealed a Stanford Type A aortic dissection extending into the right iliac artery.

IMAGING FINDINGS

- CTA Lower Extremity: Right common iliac artery occlusion, 90% right popliteal artery occlusion, no distal runoff.
- CTA Head/Neck: Dissection of ascending/descending aorta, no arch vessel involvement.
- CT Chest/Abdomen: Dissection extending to the celiac trunk, right renal artery (causing infarction), and superior mesenteric artery.

### MANAGEMENT & OUTCOME

Nicardipine infusion was initiated for BP control to prevent reflex tachycardia, maintain HR <60 bpm, and SBP 100-120 mmHg. The patient was transferred for emergent surgical repair.

### Discussion & Conclusion

Aortic dissection has an incidence of 5-30 cases per 1 million people per year, with only 15-43% diagnosed on the first ED visit. The lack of chest pain delayed consideration of aortic dissection. Important clues included:

1. Acute limb ischemia without neurological deficits elsewhere.

### 2. Severe hypertension.

### 3. Absent pulses in the affected limb.

Nicardipine was chosen for its ease of titration and ability to avoid reflex tachycardia. Prompt CT angiography is crucial for rapid diagnosis and intervention. Aortic dissection can present atypically as limb ischemia. High suspicion, early imaging, and urgent transfer to specialized care improve outcomes.

